# Construction and Validation of CRISPR/Cas Vectors for Editing the *PDS* Gene in Banana (*Musa* spp.)

**DOI:** 10.3390/cimb46120865

**Published:** 2024-12-20

**Authors:** Marcelly Santana Mascarenhas, Fernanda dos Santos Nascimento, Luana Maria Pacheco Schittino, Livia Batista Galinari, Lucymeire Souza Morais Lino, Andresa Priscila de Souza Ramos, Leandro Eugenio Cardamone Diniz, Tiago Antônio de Oliveira Mendes, Claudia Fortes Ferreira, Janay Almeida dos Santos-Serejo, Edson Perito Amorim

**Affiliations:** 1 Department of Biological Sciences, Feira de Santana State University, Feira de Santana 44036-900, BA, Brazil; marcelly.bio@hotmail.com; 2 Embrapa Mandioca e Fruticultura, Cruz das Almas 44380-000, BA, Brazil; feel.20@hotmail.com (F.d.S.N.); lucymeire.lino@gmail.com (L.S.M.L.); andresa.ramos@embrapa.br (A.P.d.S.R.); claudia.ferreira@embrapa.br (C.F.F.); janay.serejo@embrapa.br (J.A.d.S.-S.); 3 Department of Biochemistry and Molecular Biology, Federal University of Viçosa, Viçosa 36507-900, MG, Brazil; luana.schittino@ufv.br (L.M.P.S.); livia.galinari@ufv.br (L.B.G.); tiagoaomendes@ufv.br (T.A.d.O.M.); 4 Embrapa Soja, Rodovia Carlos João Strass, Londrina 86085-981, PR, Brazil; leandro.diniz@embrapa.br

**Keywords:** bananas, phytoene desaturase, CRISPR technology, vector, promoter, Prata-Anã, knockout

## Abstract

Bananas and plantains are important staple food crops affected by biotic and abiotic stresses. The gene editing technique via Clustered Regularly Interspaced Short Palindromic Repeats associated with the Cas protein (CRISPR/Cas) has been used as an important tool for development of cultivars with high tolerance to stresses. This study sought to develop a protocol for the construction of vectors for gene knockout. Here we use the phytoene desaturase (*PDS*) gene as a case study in Prata-Anã banana by the nonhomologous end junction (NHEJ) method. *PDS* is a key gene in the carotenoid production pathway in plants and its knockout leads to easily visualized phenotypes such as dwarfism and albinism in plants. *Agrobacterium*-mediated transformation delivered CRISPR/Cas9 constructs containing gRNAs were inserted into embryogenic cell suspension cultures. This is the first study to provide an effective method/protocol for constructing gene knockout vectors, demonstrating gene editing potential in a Brazilian banana variety. The constitutive (CaMV 35S) and root-specific vectors were successfully assembled and confirmed in transformed *Agrobacterium* by DNA extraction and PCR. The specificity of transformation protocols makes it possible to use the CRISPR-Cas9 technique to develop Prata-Anã banana plants with enhanced tolerance/resistance to major biotic and abiotic factors.

## 1. Introduction

Bananas and plantains are the most widely grown fruits globally owing to their socioeconomic and nutritional importance. Bananas are one of the main economic resources in several countries, particularly in South America. In 2023, Brazil ranked fifth among the world’s largest producers, with a production of 6.8 million tons contributing approximately USD 2.0 billion to the fruit agribusiness market. Regarding world production, approximately 135.1 million tons were produced and harvested on 5.9 million ha in 2023 [1].

Banana production is severely restricted by various pathogens, pests, and environmental factors that can hinder its cultivation [2,3,4]. Diseases caused by *Fusarium oxysporum* f. sp. *cubense* (Foc), *Mycosphaerella musicola*, *M. fijiensis*, *Ralstonia solanacearum*, and banana streak virus (BSV), as well as major pests such as the banana rhizome borer (*Cosmopolites sordidus*), and the nematodes *Meloidogyne* spp. and *Radopholus similis*, are major challenges for global banana and plantain production [5,6,7]. Abiotic stresses, such as water deficit and salinity, also threaten agricultural production worldwide, reducing yields and impacting plant growth, physiology, and reproduction [8,9]. Therefore, using banana varieties that are resistant to diseases, pests, water deficit, and salinity becomes one of the most effective ways to mitigate these negative impacts on fruit production [10]. In addition, bananas are parthenocarpic fruits, which makes classical genetic improvement through crosses extremely laborious since parthenocarpy implies low seed production.

Genome editing is a tool that allows the manipulation of genetic material to quickly and precisely induce mutations in regions of interest, resulting in an organism with desirable characteristics. Its application in crop plants has increased interest primarily because it simplifies regulatory steps [11].

Recent advances in new technical tools can potentially accelerate banana plant breeding to resist main biotic and abiotic factors. The banana genetic breeding program (BGBP) at Embrapa Cassava and Fruit has been investing in research that provides molecular tools to support the development of more resistant/tolerant cultivars. One of these techniques is CRISPR-Cas9 editing [12], which has been successfully used in *Musa* spp. [13,14,15].

The CRISPR/Cas9 system has been widely used in various plant species to induce mutations in the genome, allowing the study of gene functions for crop genetic improvement. This technique enables editing of genome parts by cutting, replacing, or adding sequences to the DNA of a given genotype [16,17]. Hence, editing is typically performed using plasmid vector systems that carry genes, which when integrated into the host genome, can encode the expression of the necessary products such as a nuclease, typically Cas9, and guide RNA (gRNA). Specific promoters, such as CaMV 35S for constitutive expression, or tissue-specific promoters, are also used to regulate the expression of CRISPR/Cas components. In addition, the vectors include a transformant selection marker gene, which confers resistance to antibiotics or herbicides and facilitates the identification of the transformed cells. Eventually, reporter genes such as β-glucuronidase (*GUS*) or Green Fluorescent Protein (*GFP*) are used to monitor the efficiency of the transformation [18,19,20,21].

To support the use of CRISPR/Cas9 to increase tolerance for biotic and abiotic stresses in bananas, we propose the knockout of an easily visible gene, such as phytoene desaturase (*PDS*). *PDS* is one of the limiting enzymes in carotenoid biosynthesis, and knockout of the *PDS* gene directly affects photosynthesis, which subsequently leads to albinism and plant growth retardation [13].

This albino phenotype caused by the knockout of the *PDS* gene is easy to visualize and this step, called proof of concept, is critical to start a case study and identify any bottlenecks in the use of CRISPR-Cas9 technology in plants. The proof of concept with gene editing (CRISPR/Cas9) in banana is unprecedented in Brazil, but some work has already been conducted in other countries as a way of evaluating the technique and assessing its efficiency [13,14,22]. Here, we developed an efficient CRISPR/Cas9 vector construction protocol in banana using gRNAs for the phytoene desaturase (*PDS*) gene. The two CRISPR/Cas9 constructs developed, one with a constitutive promoter and the other with a root-specific promoter, were delivered in embryogenic cell suspension cultures of the banana cultivar Prata-Anã (AAB). This is the first work with gene editing using this cultivar, which is the main banana variety planted in Brazil.

## 2. Materials and Methods

### 2.1. Plant Material

Suspensions of embryogenic cells from the male inflorescence of the banana cultivar Prata-Anã (AAB) [23] were used as explant sources for genetic transformation via CRISPR-Cas9. The cells were grown and kept in the dark at 27 ± 2 °C on an orbital shaker at 120 rpm and subcultured every 10 days for maintenance at the Plant Tissue Culture Laboratory of Embrapa Mandioca e Fruticultura, Cruz das Almas, Bahia, Brazil (12°40′48.03″ S and 39°05′20.91″ W). The Prata-Anã cultivar was selected since it is the main banana variety planted and consumed by Brazilians; however, it is susceptible to Fusarium wilt (Foc) and water deficit.

### 2.2. Identification of the PDS Gene in Banana and Design of gRNAs

To search for the *PDS* gene in bananas, the complete genome sequences of *Musa acuminata* (Ma08_t16510.2) and *Musa balbisiana* (Mba08_g16040.1) were downloaded from the SouthGreen-Banana Genome Hub database (https://banana-genome-hub.southgreen.fr/, accessed on 4 March 2024). After identifying the conserved *PDS* regions of *M. acuminata* and *M. balbisiana*, specific primers of the banana *PDS* gene [15,24] were used to rule out allelic variations in the target sites in the cultivars used in our study, and fragments of 994 bp, 2166 bp, and 332 bp of the *PDS* gene were sequenced. Genomic DNA was extracted from the leaves of the Bucaneiro (AA), Zebrina (AA), and Prata-Anã (AAB) genotypes, as described in Doyle and Doyle (1990) [25], with modifications proposed by Ferreira et al. (2019) [26]. The bands of interest were identified, selected, and purified using the PureLink^TM^ Quick Gel Extraction Kit (Thermo Fisher Scientific, Waltham, MA, USA).

The coding sequences (CDSs) from the pair-end sequencing of the *PDS* genes of the Bucaneiro (AA), Zebrina (AA), and Prata-Anã (AAB) banana genotypes were aligned using the Seqassem software version 1.0.0.0 (SeqAssm, Sequentix, Klein Raden, Germany) [27] and then aligned with the sequences of the *PDS* gene of *M. acuminata* and *M. balbisiana* using the Clustal Omega software (Clustal Omega, version 1.2.2, EMBL-EBI, Hinxton, Cambridgeshire, UK) to identify conserved regions.

Subsequently, for the design of the gRNAs, the sequences resulting from the alignment were selected based on the positioning of the PAM (Proto-spacer Adjacent Motif 5′-NGG-3) sequence; the PAM sequence is required for recognition by the Cas9 endonuclease. The gRNA off-target analysis was carried out by comparing the 20 nt gRNA target sequences in the *PDS* gene with the *M. acuminata* and *M. balbisiana* gene sequences using BLASTN (https://www.ncbi.nlm.nih.gov/, accessed on 6 March 2024) from the SouthGreen-Banana Genome Hub platform and the CRISPOR software version 5.2 (http://crispor.tefor.net/, accessed on 12 March 2024), which also took into consideration the intrinsic cleavage pattern of the Cas.

### 2.3. Construction of CRISPR/Cas9 Vectors

The construction of the vectors considered the available information on the promoter regions, terminators, and restriction enzyme sites LB (left border), 35S, U6, T7, Cas9, and RB (right border), of twelve pre-existing vectors, and the sequences that were most repeated among the selected vectors were considered to be conserved and used to assemble the new vectors, V1 and V2 (V1: with the constitutive promoter_CaMV35s and V2: *Musa* spp. root-specific promoter—Prom_*Musa*_Embrapa_005).

The first vector had a CaMV 35S promoter and four parts (Part 1 + Part 2 + Cas9 + Part 3) (Table 1). The second vector has a patented banana root-specific promoter (Prom_Musa_Embrapa_005—patent number: BR 10 2023 010195 0) and is also made up of 4 fragments (Part 1 + Part 4 + Cas9 + Part 3) (Table 1). To construct the binary plant transformation vectors containing Cas9 genes, gRNA, and the other parts, a pDIRECT-22C vector was used. The vectors were designed in the Benchling online software (version 8), available at https://www.benchling.com/crispr (accessed on 16 February 2024) to obtain vector maps and restriction enzyme predictions for digestion and validation.

The vectors were assembled using the manual Master Mix from the GeneArt™ Gibson Assembly^®^ HiFi kit (Invitrogen/ThermoFisher, Waltham, MA, USA), which allows several DNA fragments to be joined in a single isothermal reaction.

### 2.4. Transformation and Validation of CRISPR/Cas Constructs/Vectors in Escherichia coli and Assembly by Gibson Assembly

To make the *E. coli* DB3.1 and DH5-α cell competent, they were inoculated into liquid Luria–Bertani (LB) culture medium and incubated at 37 °C overnight under 150 rpm agitation. The culture was centrifuged at 5000 rpm for 5 min, the supernatant was discarded, and the pellet obtained was resuspended in 1 mL of 0.1 M CaCl_2_. After this process, the material was centrifuged at 5000 rpm for 5 min (4 °C), the supernatant was discarded, and the pellet was resuspended in 150 µL of 0.1 M CaCl_2_.

After making the cell competent, the parts of the vector were transformed into *E. coli* by heat shock. For each part, 3 µL of the vector containing the respective fragment was added to a 50 µL aliquot of competent cells, which were kept on ice for 30 min. After this period, the inoculum was subjected to a temperature of 42 °C for 45 s. The sample was immediately returned to the ice for 5 min. A quantity of 1 mL of LB medium without antibiotics was added, and the tube was incubated at 37 °C (150 rpm) for approximately 1 h. The material was then centrifuged for 5 min at 5000 rpm, and the pellet was resuspended in 50 µL of the supernatant and plated on solid LB medium plus kanamycin ( 50 mg/mL) [28].

Transformed *E. coli* cells were confirmed by extracting plasmid DNA and digesting the parts of the vectors. For Part 1, the restriction enzymes *XbaI* and *HindIII* were used. For Part 2, the *PvuII* enzyme was used, and the Cas9 was digested with *EcoRV* and the pDIRECT-22C vector with the *NheI* and *KpnI* enzymes. Digestions were carried out according to each manufacturer’s instructions. Once digested, excised, and purified, all parts were quantified and the Gibson Assembly protocol was followed. The two assembled constructs were transformed in *E. coli* DH5-α using 3 μL of the Gibson Assembly reaction and again confirmed through DNA extraction and PCR.

### 2.5. Transformation/Transfection and Validation in Agrobacterium

The protocol for transforming *Agrobacterium tumefaciens* by electroporation was adapted from Höfgen and Willmitzer (1988) [29] and standardized under the conditions described in this section.

To prepare competent cells, 1 mL of *A. tumefaciens* culture grown overnight was inoculated into 1 L of LB medium and incubated at 30 °C under agitation. Cell growth was observed until the logarithmic phase (OD_600_ 0.5–0.6), and pelleted by centrifugation at 2600× *g* for 10 min at 25 °C. The pellet was resuspended in 10 mL of ice-cold 10% glycerol and centrifuged again. A quantity of 500 µL of 10% glycerol was added to the new pellet, and the competent cells were distributed in 50 μL aliquots. The bacteria were plated on a selective LB medium containing kanamycin (50 mg/mL).

A 3 μL (100–200 ng) quantity of plasmid DNA extracted from transformed *E. coli* DH5-α was added to 50 μL of *Agrobacterium* competent cells and gently homogenized to transform the two constructs. The cells were transferred to an electroporation cuvette (2 mm) and taken to the electroporator, where 2.5 kV, 25 µF, and 400 Ω pulses were applied. A 1 mL Super Optimal Medium with Catabolic Repressor (SOC) was immediately added. The material was transferred to microtubes and kept on a shaker at 200 rpm for 2–3 h at 30 °C. Subsequently, it was centrifuged and resuspended in 100 μL of its supernatant, plated on solid LB medium with kanamycin (50 mg/mL), and kept at 28 °C for 42–72 h. Confirmation of *Agrobacterium* transformant cells was carried out via plasmid DNA extraction and PCR using vector-specific primers.

### 2.6. Confirmation of the Integration of CRISPR/Cas Constructs

For plasmid DNA extraction, 5 mL of the *Agrobacterium* inoculum was centrifuged at 10,000 rpm for 2 min, the supernatant was discarded, and 200 μL of ice-cold solution I (Table S1) and 10 mg/mL RNAse at 10% *v*/*v* were added. The material was incubated for 10 min at room temperature. After this period, 200 μL of ice-cold solution II (Table S1) was added. The tubes were gently homogenized and kept on ice for 5 min. Subsequently, 200 μL of ice-cold solution III (Table S1) was added, and the tubes were gently homogenized and kept on ice for 5 min. The material was centrifuged at 10,000 rpm for 10 min, and 400 μL of the supernatant was collected and poured into new microtubes. Briefly, 800 μL of ice-cold isopropanol was added and homogenized, and were samples kept at −20 °C for at least 1 h.

After this period, it was centrifuged at 10,000 rpm for 15 min, and the supernatant was discarded. Ice-cold 70% ethanol (500 µL) was added to the pellet, the sample was centrifuged at 10,000 rpm for 15 min, and the supernatant was discarded. The pellet remained at room temperature (or in a dry bath at 45 °C), followed by resuspension in approximately 20 μL of nuclease-free water and kept in a freezer (−20 °C). DNA quantification was performed on Quibit and 1% agarose gels in TAE 1X (adapted from Sambrook et al., 1989 [28]).

Plasmid DNA extracted from *Agrobacterium tumefaciens* transformed with constructs 1 and 2 was subjected to PCR using primers Vc9_Fw and Vc9_Rv, as well as Vp3C9_Fw and Vp3C9_Rv (Table 2), to confirm Parts 1, 2, 3, 4, vector, and Cas9.

The samples were amplified in a Veriti thermal cycler (Applied Biosystems, Waltham, MA, USA) with programming adapted from Cellco (Cellco Inc., Germantown, MD, USA). The primer sequences and annealing temperatures are shown in Table 2. The amplification products were separated through electrophoresis on a 1% agarose gel at 70 V in TAE buffer for 45 min in ethidium bromide. They were then visualized and photographed under ultraviolet light on an L-Pix Touch documentation system (Loccus, Cotia, Brazil).

### 2.7. Delivery of the CRISPR/Cas9 Plasmid to Prata-Anã Cells in Suspension

The plant embryogenic cell suspension culture (ECS) was diluted to 33% (*v*/*v*) in liquid Dichlorophenoxyacetic acid (2,4-D) medium supplemented with acetosyringone (AS) 200 μM. The four treatments were arranged in 24-well plates. For treatment 1 (T1), a 200 μL plant cell (PC) and 1 mL liquid 2,4-D culture medium were added. For treatment 2 (T2), 200 μL of PC and 1 mL of *Agrobacterium* (OD_600_) of 1.2 units, with empty vector were added. Treatment 3 (T3) involved addition of 200 μL PC and 1 mL of *Agrobacterium* transfected with construct 1 (V1_CaMV35s), whereas treatment 4 (T4) involved addition of 200 μL PC and 1 mL of *Agrobacterium* with construct 2 (V2_Musa_root specific promoter).

The plates were incubated for 6 h at 25 °C and 25 rpm on a rotary shaker in the dark. The mixture of plant and bacterial cells was transferred to a sterile 50 μm polyester mesh (4 cm^2^) deposited on three sterile filter papers to remove excess liquid medium. The polyester membrane containing the cells was transferred to co-cultivation plates containing 10 mL of solid 2,4-D medium (pH 5.3) supplemented with AS and incubated at 26 °C in the dark for 6 days.

After this period, the membranes were transferred to plates with solid 2,4-D culture medium supplemented with timentin (200 mg/L) and kanamycin (50 mg/mL), and incubated in the dark at 25 ± 2 °C for 30 days. The membranes were then transferred to BAP (6-benzylaminopurine) + AIA (indoleacetic acid) medium, plus timentin and kanamycin, where they remained for 15 days in the dark at a temperature of 25 ± 2 °C. The plates were transferred to a 16 h/8 h photoperiod at 25 °C and subcultured every 30 days if necessary.

## 3. Results

### 3.1. Design of the gRNAs

**Figure 1:**
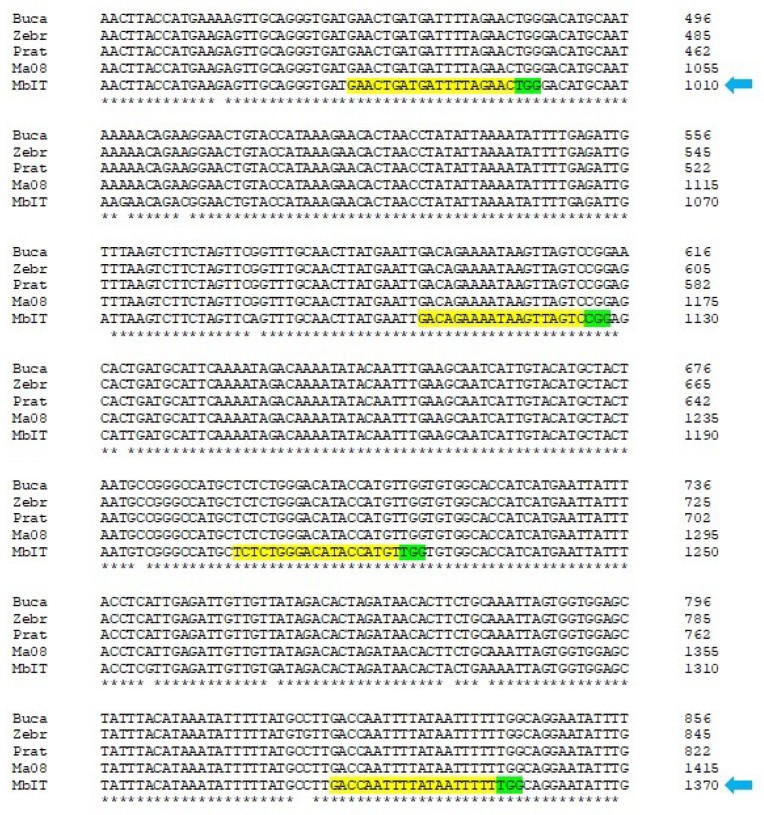
Partial alignment resulting from the sequencing of the *PDS* genes of the banana genotypes Bucaneiro (AA), Zebrina (AA), and Prata-Anã (AAB) and sequences of the *PDS* gene of *Musa acuminata* and *Musa balbisiana* for gRNA design. The blue arrows indicate the selected gRNAs. Yellow highlights represent the gRNA nucleotides and green highlights indicate the PAM sequences. * Correspondence between the compared sequences, indicating a high degree of conservation.

By aligning the CDS resulting from the sequencing of the *PDS* genes of the Bucaneiro (AA), Zebrina (AA), and Prata-Anã (AAB) banana genotypes with the *PDS* gene sequences of *M. acuminata* (Genome A) and *M. balbisiana* (Genome B), the gRNAs could be selected. The off-target activity of the four gRNAs was assessed using BLASTN on the SouthGreen-Banana Genome Hub platform. All the gRNAs showed 95% nucleotide homology with the banana *PDS* gene (Figure 1).

Two gRNAs were selected (indicated by blue arrows) to maximize the chances of mutations and deletion of large fragments as the gRNA targets two different exons of the *PDS* gene in Prata-Anã, which contains both genomes (genome A and B). gRNA1 (GAACTGATGATTTTAGAACTGG) and gRNA2 (GACCAATTTATAATTTTTTGG) were integrated into the constructs.

### 3.2. Construction of CRISPR/Cas Vectors

The parts of the constructs (Part1_LB_U6pro_sgRNABsaI_U6term; Part2_35s_T7_promotor_const; Part3_OCSterm_RB; Part4_35s_T7_promotor; Cas9) were synthesized, the sequences in common with various vectors were compared, and those with the highest level of conservation were selected to create the new vectors.

**Figure 2:**
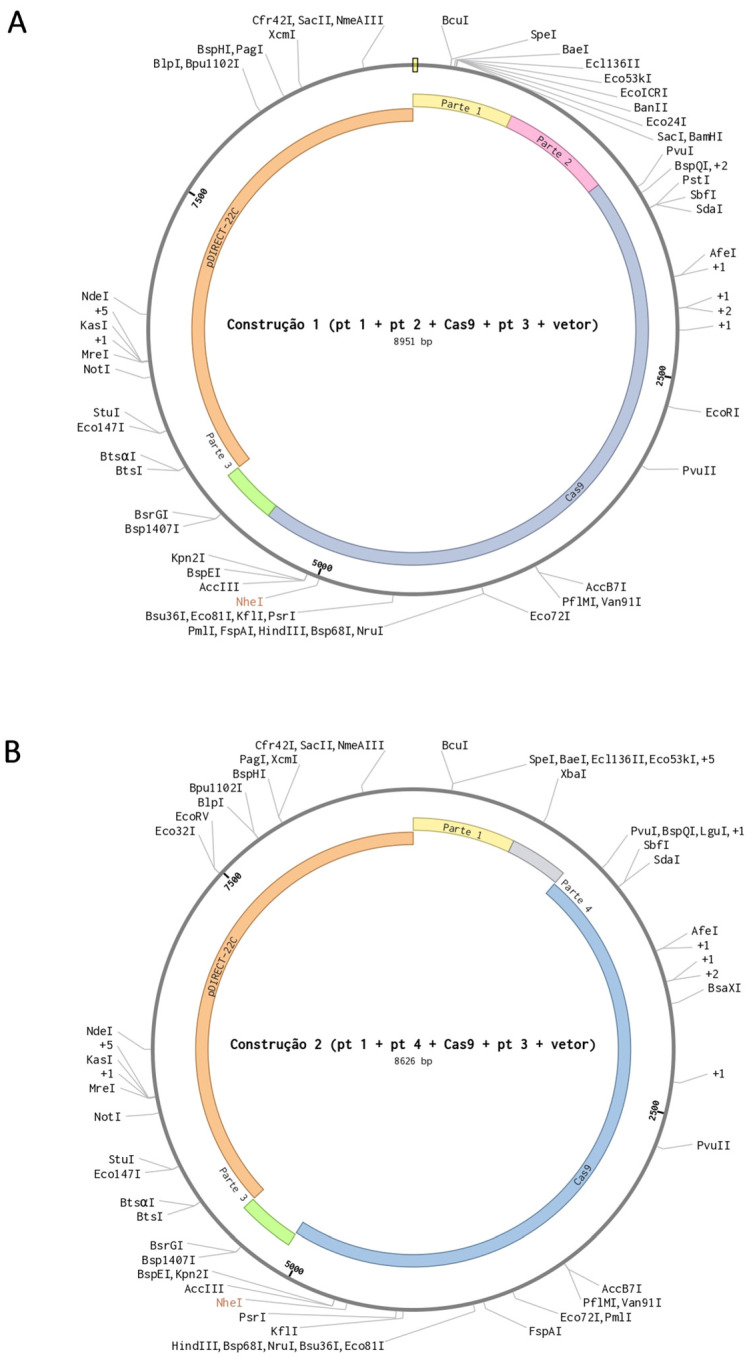
Maps of the vectors with CaMV 35S promoter and Part 1 + Part 2 + PCR Cas9 + Part 3 + Vector pDIRECT-22C (**A**) and vector with root-specific promoter with Part 1 + Part 4 + PCR Cas9 + Part 3 + Vector pDIRECT-22C (**B**) for use in banana cisgenesis via *Agrobacterium* transformation. The outer part of the vector contains the possible restriction enzymes for digesting the vectors.

After selecting the sequences and constructing the parts, the maps of the vectors (Figure 2) for gene editing in bananas via *Agrobacterium* transformation were drawn up.

### 3.3. Standardization of Protocol for Processing and Digestion of Parts

**Figure 3:**
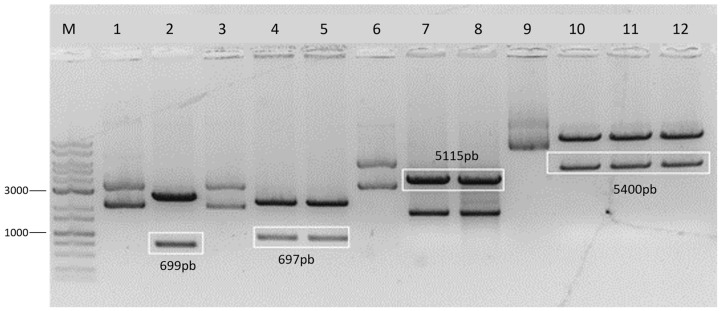
Digestion of the parts and vectors of interest in Gibson Assembly. M: 1 kB Plus NeoBio molecular weight marker (CV-1000 kB); 1: miniprep from Part 1 (undigested control); 2: 699 bp fragment excised from Part 1; 3: miniprep from Part 2 (undigested control); 4 and 5: 697 bp fragments excised from Part 2; 6: Cas9 miniprep in pUC57 (undigested control); 7 and 8: 5115 bp fragments excised from Cas9; 9: pDIRECT-22C vector miniprep (undigested control); 10, 11, and 12: 5400 bp fragments excised from pDIRECT-22C.

The competence of *E. coli* DB3.1 and DH5a and transformation by heat shock were standardized and confirmed by DNA extraction and digestion of the parts. Parts 1, 2, 3, and 4, and Cas9, were transformed into *E. coli* DH5-α and pDIRECT-22C into *E. coli* DB3.1 to multiply the plasmids. The transformant colonies underwent a plasmid DNA extraction process (miniprep). The Part 1 miniprep was digested with the restriction enzymes XbaI (Cellco) and HindIII (Promega, Madison, WI, USA), and a 699 bp fragment was released (Figure 3). The Part 2 miniprep was digested with the restriction enzyme PvuII (Jena Bioscience, Jena, Germany), releasing a fragment of 697 bp (Figure 3).

Cas9 is one of the essential parts for Gibson Assembly of the vector and is largely responsible for the functioning of the CRISPR/Cas system. After transformation and selection in a medium containing the antibiotic kanamycin (50 mg/mL), plasmid DNA was extracted and digested by the EcoRV restriction enzyme, resulting in the release of a 5115 pb fragment (Figure 3).

The pDIRECT-22C vector is also an important element for assembling the vector of interest by Gibson Assembly, as it contains overlap and is widely used for gene expression in plants. This vector contains a gene for resistance to the antibiotic kanamycin. Therefore, it was also transformed into *E. coli* DB3.1 and selected in a culture medium containing the antibiotic kanamycin (50 mg/mL). Plasmid DNA was extracted, and the resulting sample was digested using the restriction enzymes NheI and KpnI (Cellco), releasing a 5400 bp fragment (Figure 3), which, like the other fragments, was excised and purified.

Parts 3 and 4 were not cloned in the vector; hence, digesting and purifying them was not necessary; they were just resuspended in 20 μL of nuclease-free water to a final concentration of 25 ng/μL.

### 3.4. Quantification of Vector Parts and Assembly by Gibson Assembly

Parts 1, 2, 3, and 4, previously digested, excised, and purified Cas9, and pDIRECT-22C, were assayed by fluorimetric quantification using a Qubit 4 fluorometer according to the manufacturer’s protocol.

The assembly protocol suggests a concentration of 0.08 pmols of each of the inserts in the reaction. To assemble the Gibson Assembly, the necessary amount, in μL, of each part was calculated, combining these fragments in a single enzymatic reaction. Accurate quantification and the use of the correct molar proportions between the fragments and the vector are critical for the efficient assembly by Gibson Assembly.

### 3.5. Transformation and Confirmation of Constructs in Agrobacterium Tumefaciens

**Figure 4:**
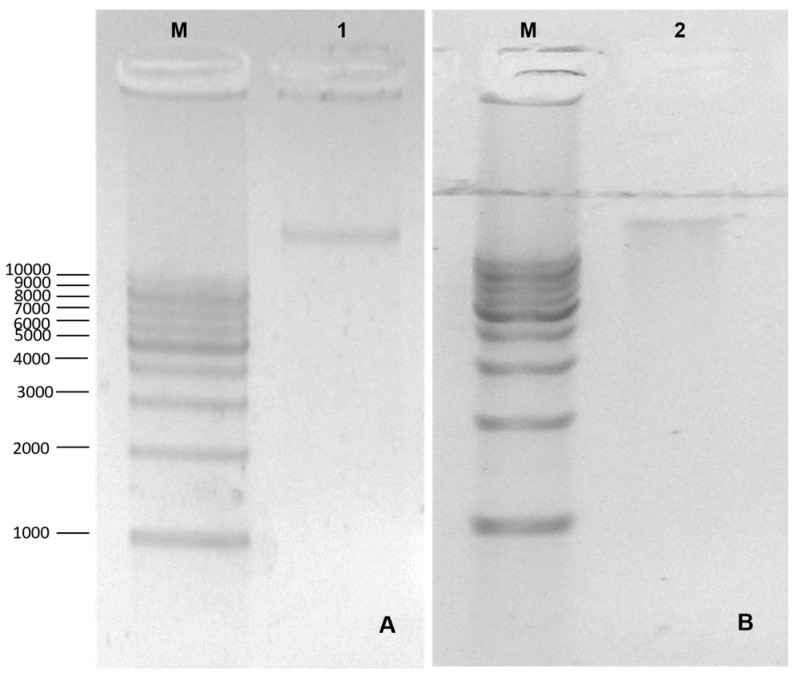
Extraction of plasmid DNA from the constructs inserted into *Agrobacterium tumefaciens*. M: molecular weight marker 1 kB DNA Ladder—MMK-105S (Cellco); 1 and 2: amplicons with more than 10,000 bp equivalent to construct 1 (**A**) and 2 (**B**).

**Figure 5:**
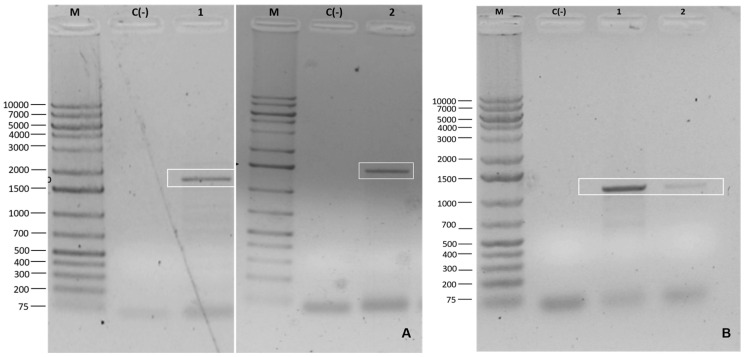
PCR amplification of the *Agrobacterium tumefaciens* strain transformed with constructs 1 and 2. (**A**) PCR with primer set VC9_Fw and VC9_Rv (M: molecular weight marker 1 kB Plus DNA Ladder—MMK-130S (Cellco); C(-): negative control of the reaction with water; 1 and 2: 1912 bp amplicons at 45 °C for constructs 1 and 2). (**B**) PCR for primers Vp3C9_Fw and Vp3C9_Rv; M: marker; C(-): negative control; 1 and 2: 1000 bp amplicons at 51 °C.

After standardizing and confirming the protocol developed, the two assembled constructs (V1_CaMV35s and V2_Musa_Root) were transformed into *Agrobacterium* using approximately 3 μL (100–200 ng) of plasmid DNA extracted from the transforming *E. coli* strains previously selected for kanamycin resistance and through the electroporation transformation protocol. The constructs were confirmed through DNA extraction (Figure 4) and PCR (Figure 5).

### 3.6. Delivery of the CRISPR/Cas9 Plasmid in Banana cv. Prata-Anã

**Figure 6:**
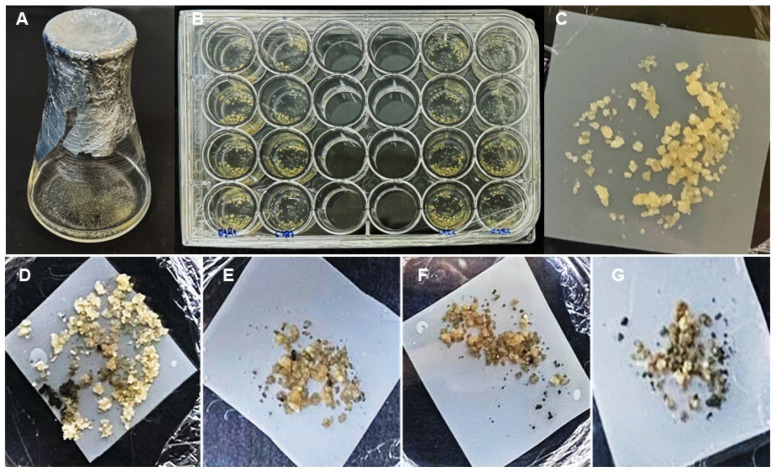
Process of genetic transformation and regeneration of the Prata-Anã banana. (**A**) Embryogenic cell suspension used for transformation with the CRISPR/Cas9 plasmid; (**B**) embryos in 2,4-D culture medium and transforming *Agrobacterium*; (**C**) embryos in polyester membrane and 2,4-D + AS culture medium (T1) after 0 days and the different treatments (**D**–**G**) with BAP + AIA + Kan + Timentin culture medium ((**D**): T1, (**E**): T2, (**F**): T3, (**G**): T4) after 70 days of transformation, kept at 25 °C with a 16 h/8 h photoperiod.

Mutants were generated by delivering the two CRISPR/Cas9 constructs (V1_CaMV35s and V2_Musa_Raiz) into a suspension of embryogenic cells from the Prata-Anã (AAB) cultivar using transformation via *Agrobacterium*. The treatments were organized into 24-well plates, with treatments comprising the following: T1 consisting of the plant cell (PC) and liquid 2,4-D culture medium; T2 consisting of PC and empty vector; T3 consisting of PC and Construction 1; and T4 consisting of PC and Construction 2 (Figure 6).

The germinating plant cells were transferred to jars or test tubes with solid BAP + AIA medium for regeneration.

## 4. Discussion

Banana production is severely affected by biotic and abiotic factors. Among the biotic factors, Fusarium wilt (STR4 and TR4) is the most destructive. Considering abiotic factors, water deficit, also caused by climate changes, not only leads to losses in production, but also contributes to intensify biotic stresses already at play [30]. Given this scenario, adequate technologies are essential to develop new banana genotypes endowed with durable and broad-spectrum tolerance or resistance. Traditional breeding cannot always produce new genotypes that deal with rapid changes. In the case of banana plants, classical breeding is even more time-consuming owing to the inherent parthenocarpy of the species, which leads to low seed production.

Gene editing technology makes it possible to precisely and quickly modify the target DNA at a specific location, inducing specific genetic variations but maintaining the genetic identity of elite cultivars [31,32]. Developing well-defined methods and protocols using this technology allows reproducibility, consistency and reliability, versatility, minimization of editing errors, facilitation of systems delivery to the site of interest, and promotion of resource savings.

CRISPR/Cas vector systems are well-designed genetic constructs made up of a Cas9 enzyme or its variants, such as Cas12a (Cpf1), Cas13, and others, responsible for cleaving the DNA and RNA respectively at the site of interest, which allows for precise editing, as well as a guide RNA that directs the enzyme to the target of the mutation. The system also includes promoters that can be constitutive, tissue-specific, or inducible; insertion sequences, selection markers, cloning, and replication elements are also part of this system [33,34,35].

The 35S promoter from the cauliflower mosaic virus (CaMV 35S) is widely used in plants to ensure the continuous expression of genes that regulate all cell types. Its application is well reported in several crops such as rice [36,37,38,39], tomato [40,41], and soybean [42,43], with successful cases for the expression of Cas9. However, tissue-specific promoters are activated only in the designated tissues or at specific moments in development, with precise regulation of expression, reducing the chances of off-target mutations and the involvement of other tissues [44,45].

Krasnyanski et al. (2001) [46] used two promoters derived from a binary vector to transform tomatoes. The *uidA* gene was driven by the CaMV 35S/AMV or the E-8 promoter specific for fruit ripening. The authors successfully expressed the gene with both promoters, and the specific promoter was not expressed in other tissues, only in the fruit.

Although the CaMV 35S promoter is widely used, growing efforts are needed to explore tissue-specific promoters to achieve more efficient and targeted expression. This study developed two vectors using the CaMV 35S promoter and the root-specific promoter (Prom_Musa_Embrapa_005—patent number: BR 10 2023 010195 0). The root-specific promoter only activates the transcription of genes locally in root cells, ensuring that their essential functions, such as nutrient and water absorption, hormone production, substance transport, food storage, interaction with microorganisms, and other functions, are performed optimally [33,47]. This type of promoter can act by recognizing specific regulatory elements, interacting with transcription factors, environmental signals, or tissue restriction [48,49].

Diseases such as *Fusarium* wilt and nematodes are transmitted through the soil. Using a root-specific promoter associated with the CRISPR/Cas system offers an advantage for increasing disease resistance in plants as it is a more precise and efficient approach to the local defense response. When the system is activated in the roots, genes related to stress signaling or the synthesis of antimicrobial compounds, such as phytoalexins and specific proteins, as well as plant hormones, can be expressed and enable the development of localized resistance, minimizing off-target effects on other plant structures [33,44,48].

In soybean, the GmADR1 promoter was used to direct the expression of the GmCaM4 gene in root tissues, and the plants showed high resistance to salinity [44]. The maize *Chitinase A1* and *Phospholipid transferase* promoters (pZmCTA1 and pZmPLTP) were used to design a specific callus system with beneficial effects on hereditary mutations and a reduction in somatic mutations [50].

In Arabidopsis and soybean, the GmPRP2 promoter was tested for its expression pattern, and it was concluded that this is a preferential root promoter and can be used to improve functions related to plant roots, such as nutrition, tolerance, or resistance to biotic and abiotic stressors [51]. The TIP2 promoter and 18 other promoter sequences were evaluated for specificity in banana and *Nicotiana tabacum* (tobacco) roots. These promoters could be used in new CRISPR/Cas constructs, expanding the options for controlling diseases, pests, and other stresses [52].

The use of specific promoters in the CRISPR/Cas system has been explored and their successful integration into the CRISPR/Cas system has been confirmed; the choice of promoters depends on the tissue to be studied and the objective of the experiment [53,54]. The authors successfully confirmed the integration of promoters into a CRISPR/Cas9 system to facilitate research focused on plant breeding using specific or constitutive promoters for a better understanding of the mechanisms surrounding the expression of genes of interest and for validation using transformed plants.

CRISPR/Cas vectors allow the efficient delivery of essential elements such as the Cas protein, gRNA, and other components. The Cas9 protein belongs to type II class II and was developed from *S. pyogenes*. Cas9 nucleases are guided by CRISPR RNAs (crRNAs), which resemble transactivating crRNAs (tracrRNAs) and facilitate the formation of the ribonucleoprotein complex [34]. However, most Cas9 genome editing applications use the gRNA molecule, designed by fusing crRNA and tracrRNA into a single RNA molecule. In most cases, CRISPR/Cas9 requires a target site of 17 to 20 base pairs (bp) directly adjacent to a 5’-NGG PAM sequence (motif adjacent to the protospacer) to be effectively recognized by the gRNA, as used in this study [55,56].

Promoters control the delivery of these genes, increasing the precision of gene editing. Different vectors can be found for different organisms. pCAMBIA is a binary vector that is widely used owing to its versatility, high efficiency of *Agrobacterium*-mediated transformation in mono- and dicot species, and easy manipulation in the laboratory [14,57]. The authors analyzed several vectors already available in the literature to construct the final vector. The regions to make up the new vector were chosen based on the degree of homology between the nucleotide sequences of each structure. pCAMBIA was found in most of the works and has a high degree of conservation, which justifies its use.

In the present study, the cloning process with the pDIRECT-22C vector and genetic construction was carried out in *E. coli.* Once Gibson Assembly had assembled the DNA vector in an in vitro reaction, the plasmids were transferred to *A. tumefaciens*. This vector is often used for cloning, as it supports multiple gRNAs and multiplex manipulation of genes. It also has antibiotic resistance genes such as kanamycin and ampicillin, for selection of transformed cells with the vector insert [58,59].

Various plant transformation methods can be used to apply the CRISPR/Cas system. The most common is transformation mediated by *A. tumefaciens*, a bacterium capable of transferring its genetic material into the plant genome. The method has high efficiency, stability in delivering transgenes, less physical damage to DNA, and can be used in different species. However, its application in monocots is limited [57,60,61].

Explants are small fragments of living tissue that can be removed from different parts of a plant, such as roots, stems, and leaves, and play an important role in the efficiency of transformation [62]. In bananas, stem apices, meristems, and male inflorescences can be used as a source of explants. In this study, we chose to use embryos from male inflorescences due to the easy regeneration of transformed plants [63,64]. When compared to other types of explants, such as leaves or roots, these cells offer a higher transformation rate and a lower frequency of somaclonal mutations due to their greater totipotency capacity [65,66,67]. The efficiency of *Agrobacterium*-mediated transformation in embryogenic cells can be attributed to their greater cellular competence, allowing effective integration of the transferred DNA and resulting in more viable mutants [68,69]. These data suggest that, although different explants can be successfully used in genetic transformation, embryogenic cells represent a superior option for transformation efficiency and plant regeneration, especially for recalcitrant species.

Knockout of the *PDS* gene in banana plants and other crops using CRISPR/Cas9 technology has been widely studied due to its function as a phenotypic marker of successful editing. Disruption of this gene causes a loss of function in carotenoid biosynthesis, resulting in albino plants, which allows direct assessment of transformation efficiency [15,42]. Studies conducted on Cavendish banana cultivars used embryogenic cell suspensions to knock out the *PDS* gene, resulting in a high regeneration efficiency and expression of the albino phenotype [14]. These findings reinforce the importance of properly selecting explants to maximize the success of transformation and gene editing via CRISPR/Cas in plants.

The development of an efficient protocol for constructing CRISPR/Cas9 systems using constitutive promoters (such as CaMV 35S) and tissue-specific promoters, followed by *A. tumefaciens*-mediated transformation and knockout of the *PDS* gene in banana, represents a significant advance in plant biotechnology. This protocol allows precise gene editing, with the *PDS* gene acting as a visual marker to confirm the effectiveness of the editing as its interruption results in an albino phenotype in the plants.

Selecting explants with a high regenerative capacity is essential to ensure efficient integration of the CRISPR/Cas vector and the regeneration of viable plants. Using constitutive promoters ensures broad and continuous expression of the Cas9 endonuclease throughout the plant. In contrast, specific promoters can regulate expression in target tissues, offering high precision in the edits. This approach highlights the importance of combining a robust vector construct with the careful selection of explants and promoters, resulting in an efficient methodology for plant breeding.

## 5. Conclusions

In this study, we developed a vector construction protocol, and validated a construct/cassette as a biotechnological product, for knockout of the *PDS* gene in Prata-Anã banana, using CRISPR/Cas9 technology. This study enables the continuation of recent research focused on the genetic improvement of bananas against biotic and abiotic stressors, such as Fusarium wilt and water deficit. The methods used so far to edit genes using CRISPR/Cas9 technology have proved successful in various crops. The consolidation of a constitutive and root-specific vector/promoter and the possibility of knocking out the *PDS* gene (proof of concept) in the Prata-Anã cultivar is unprecedented in Brazil. The vectors developed here will be used in future studies to knock out genes for resistance/tolerance to biotic and abiotic stresses in banana varieties of commercial interest. However, challenges such as variability in transformation efficiency, possibility of off-target effects, and limitations imposed by specific PAM sequences still need to be overcome. In the future, the integration of new endonuclease variants and accessory tools may improve the performance of the technique. This protocol can be used to optimize other methods of delivering the components of the CRISPR/Cas system, such as biobalistics, ribonucleoproteins (RNPs), and protoplasts (transfection/electroporation), and can be used in vegetative propagated species for resistance/tolerance to biotic and abiotic factors. It also may have a significant impact on genetic improvement in agriculture by addressing the challenges of global food security.

## Figures and Tables

**Table 1:** Composition of the parts used in constructing the CRISPR/Cas 35S vectors and the specific banana root promoter.

Parts	Construction of CRISPR/Cas9 Vectors CRISPR/Cas9
	Composition
Part 1	LB_U6 promoter_gRNA BsaI_U6 gene termination signal
Part 2	35s_T7_promoter_constitutive (gRNA with U6 promoter)
Part 3	OCS 3′ terminator + RB
Part 4	35s + T7 + gRNA with U6 promoter (promoter banana)
Cas9	Bba_K1218011

LB = left border, RB = Right border, U6: RNA polymerase III; T7: expression—gRNA, OCS: terminator.

**Table 2:** Specific primers for confirmation of constructs 1 and 2 assembled by Gibson Assembly after transformation in *Agrobacterium tumefaciens*. C1: construct 1 and C2: construct 2.

Name	Seq 5′-3′	pb	%GC	Ta	Amplicon
Vc9_Fw	CTACCCTCCGCGAGATCATC	20	60%	45 °C	1912 pb C1
Vc9_Rv	CGACCTCATCCACAATGTTGC	21	52.4%	45 °C	1587 pb C2
Vp3C9_Fw	GCGTTACCTTCCAAATACGTG	21	47.6%	51 °C	988 pb
Vp3C9_Rv	CGCACGGTGAAACAGAAC	18	55.6%	51 °C	988 pb

## Data Availability

The original contributions presented in this study are included in the article/Supplementary Materials. Further inquiries can be directed to the corresponding author(s).

## Supplementary Materials

The following supporting information can be downloaded at: https://www.mdpi.com/article/10.3390/cimb46120865/s1, Table S1. Preparation of could solutions.
