# Supplementary material for: Construction and Validation of CRISPR/Cas Vectors for Editing the PDS Gene in Banana (Musa spp.)
Source: Curr Issues Mol Biol. 2024 Dec 20;46(12):14422–37. doi: 10.3390/cimb46120865 (PMC11674420; doi:10.3390/cimb46120865)
Supplement: Supplementary file 1 [file cimb-46-00865-s001.zip › cimb-3272783-supplementary.pdf]

Supplementary materials

Table S1. Preparation of could solutions.

Solution 1	Solution 2	Solution 3
Tris HCl 1M; pH 8.0; 25mM (0.25 mL);	Distilled water (8.0 mL);	Potassium acetate 5M; pH 5.5 (29.45g/60 mL of H_2O);
EDTA 0.5M; pH 8.0; 10mM (0.2 mL);	NaOH 2M (1.0 mL);	Acetic acid (11.5 mL);
Glucose 500mM; 50mM (1.0 mL);	SDS 10% (1.0 mL).	Destilled water (28.5 mL);
Destilled water (8.55 mL);		pH 5.5.
pH 8.0;		
Autoclave for 20 minutes.		
